# Fluorescent “Turn-Off” Detection of Fluoride and Cyanide Ions Using Zwitterionic Spirocyclic Meisenheimer Compounds

**DOI:** 10.3390/molecules22111842

**Published:** 2017-10-27

**Authors:** Marina Benet, Marc Villabona, Carles Llavina, Silvia Mena, Jordi Hernando, Rabih O. Al-Kaysi, Gonzalo Guirado

**Affiliations:** 1 Departament de Química, Universitat Autònoma de Barcelona, E-08193 Bellaterra, 08193 Barcelona, Spain; marina.benet@e-campus.uab.cat (M.B.); marc.villabona@uab.cat (M.V.); carlesllavina2@gmail.com (C.L.); Silvia.Mena@uab.cat (S.M.); 2 College of Science and Health Professions-3124, King Saud bin Abdulaziz University for Health Sciences/King Abdullah International Medical Research Center, Ministry of National Guard Health Affairs, 11426 Riyadh, Saudi Arabia

**Keywords:** spirocyclic, Meisenheimer complex, fluoride, cyanide, colorimetric, fluorescence, zwitterionic

## Abstract

Stable zwitterionic spirocyclic Meisenheimer compounds were synthesized using a one-step reaction between picric acid and diisopropyl (ZW1) or dicyclohexyl (ZW3) carbodiimide. A solution of these compounds displays intense orange fluorescence upon UV or visible light excitation, which can be quenched or “turned-off” by adding a mole equivalent amount of F^−^ or CN^−^ ions in acetonitrile. Fluorescence is not quenched in the presence of other ions such as Cl^−^, Br^−^, I^−^, NO_2_^−^, NO_3_^−^, or H_2_PO_4_^−^. These compounds can therefore be utilized as practical colorimetric and fluorescent probes for monitoring the presence of F^−^ or CN^−^ anions.

## 1. Introduction

**Scheme 1:**
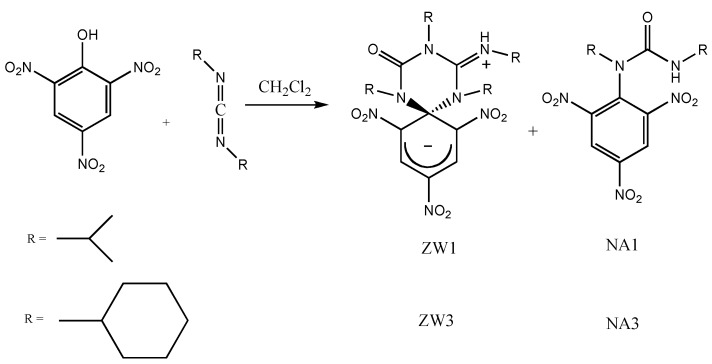
Synthesis of ZW1 and ZW3. The reaction produces NA1 and NA3 as side products.

Spirocyclic compounds have found use in the fields of organic optoelectronics [1,2,3], photochromism [4,5], and medicinal chemistry [6,7,8]. The synthesis of most spicrocyclic compounds involves several steps with tedious product separation and purification. Our group has developed the facile synthesis of an exceptionally stable and fluorescent zwitterionic spirocyclic Meisenheimer compound (ZW1) via a one-step reaction between picric acid and diisopropyl carbodiimide [3,9,10] (reaction Scheme 1). The molecular structure of ZW1 was characterized via X-ray crystallography [10], revealing its zwitterionic nature.

**Scheme 2:**
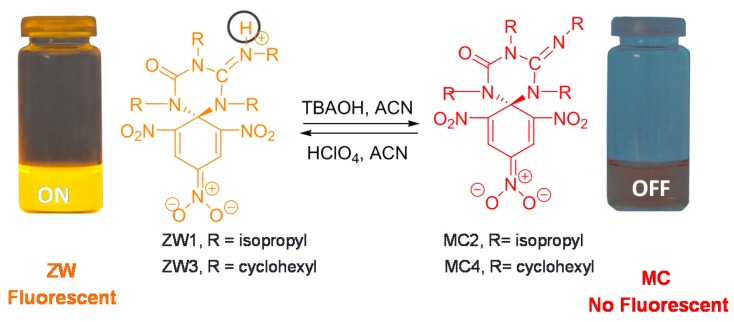
Fluorescence switching (on = ZW/off = MC) of ZW upon addition of base and acid.

Compound ZW1 is composed of two main structural fragments: (i) a 1,3,5-trinitrocyclohexadiene anion chromophore displaying intense orange light emission; and (ii) a triazine ring with a labile iminium proton. The relevant features of these two building blocks prompted us to explore the potential use of ZW1 as a colorimetric and fluorescent chemosensor. In a previous work, we demonstrated the reversible fluorescence (on/off) switching of ZW1 upon deprotonation using tetrabutylammonium hydroxide (TBAOH) as a base in acetonitrile (ACN) [11]. This behavior resulted from emission quenching in the Meisenheimer complex (MC2, Scheme 2) formed upon base addition, which was ascribed to photoinduced electron transfer from the deprotonated imine group to the photoexcited 1,3,5-trinitrocyclohexadiene anion fluorophore. Importantly, this process was inhibited upon protonation with perchloric acid to regenerate the zwitterionic state of the system, thus demonstrating that ZW1 acts as a fluorescent pH-sensor (Scheme 2) [11].

Based on these results, we aim herein to expand the use of ZW1 and analogue compound ZW3 for the chemosensing of other relevant analytes such as F^−^ and CN^−^ anions. In this sense, there are several promising anion chemosensors based on colorimetric changes or change in fluorescence. These gadolinium (III) organometallic complex-based sensors found promising use for in vivo imaging and fluorescence detection of biologically harmful fluoride ions [12,13,14]. Note that excess fluoride ions in biological fluids and tissues can lead to fluorosis and osteoscaroma, whereas a fluoride deficiency causes osteoporosis and poor dental health [15]. Another significant anion to detect is the cyanide anion, which is toxic towards humans and the environment [16]. In this sense, extensive methods and probes have been developed for the qualitative and quantitative detection of these anions [14,17,18,19,20,21].

In this article, we study the colorimetric and fluorescence response of ZW1 and its analogue ZW3 in the presence of CN^−^ and F^−^ anions, as well as the interference caused by other ionic species such as Cl^−^, Br^−^, I^−^, H_2_PO_4_^2−^, NO_3_^−^, and NO_2_^−^. 

## 2. Results

### 2.1. Synthesis and Photophysical Properties of ZW1 and ZW3

The synthesis of ZW1 was previously reported by our group (Scheme 1) [9,10]. An improved method for the preparation of this compound is described in the experimental section, which ameliorates the experimental conditions previously used. Briefly, *N*,*N′*-diisopropylcarbodiimide and picric acid are mixed in a 2:1 mole ratio in dry acetonitrile and allowed to react for several hours at 60 °C. The desired product ZW1 was obtained together with the monosubstituted compound NA1, which were isolated using flash column chromatography. We also report the synthesis of a similar compound (ZW3) bearing different substituent groups (R = cyclohexyl) in its triazine ring, which was obtained following a similar procedure using *N*,*N′*-dicyclohexylcarbodiimide instead. The synthesis, purification, and characterization of this compound are detailed in the experimental section. These compounds, either as crystalline solids, nanoparticles suspended in water [22], or as solutions in aprotic solvents, are very stable at room temperature and under ambient light conditions. Samples of ZW1 that were synthesized a decade ago did not show any signs of degradation as confirmed by ^1^H-NMR spectra.

**Figure 1:**
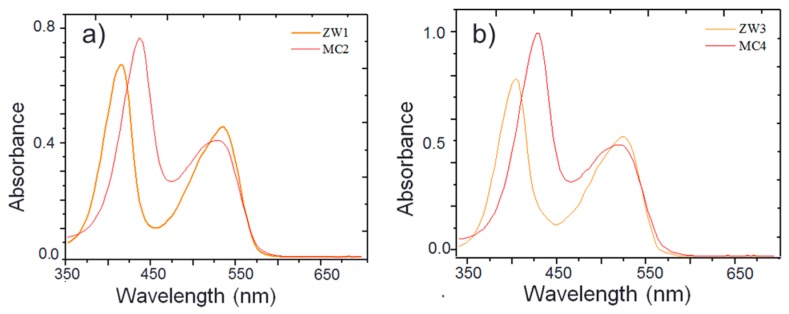
(**a**) Absorption spectra of ZW1 in acetonitrile and its corresponding deprotonated MC2 compound (c = 3.85 × 10^−5^ M); (**b**) Absorption spectra of ZW3 in acetonitrile and its corresponding deprotonated MC4 compound c = 7.69 × 10^−5^ M).

Replacing the isopropyl groups in ZW1 with cyclohexyls in ZW3 does not affect the overall shape of the absorption spectrum of their 1,3,5-trinitrocyclohexadiene anion chromophore (Figure 1a,b). However, large changes in molar extinction coefficients (ε) were observed. For ZW1, the ε_(406 nm)_ = 16,600 cm^−1^ M^−1^ and for ZW3, ε_(406 nm)_ = 10,810 cm^−1^ M^−1^ at the absorption maximum. In addition, a subtle increase in fluorescence quantum yield (ϕ_F_) was encountered: ϕ_F_ = 0.5 and 0.6 for ZW1 and ZW3, respectively. This 20% increment in ϕ_F_ for ZW3 might be attributed to the bulkier cyclohexyl groups in its triazine ring, which may shield the electron density on the adjacent nitrogen atoms and, as such, increase their oxidation potential and disfavor fluorescence quenching of the 1,3,5-trinitrocyclohexadiene anion chromophore via photoinduced electron transfer. Finally, the presence of three additional methylene groups per substituent in the triazine moiety of ZW3 increases its solubility in non-polar solvents and its miscibility with polymeric matrices such as polyvinyl alchohol and polymethyl methacrylate. These enhanced properties will be useful for future applications involving these derivatives.

In terms of chemical properties, compound ZW3 was found to preserve the colorimetric and fluorescent pH-response previously reported for ZW1 [11]. Adding a base such as tetrabutylammonium hydroxide to an acetonitrile solution of ZW3 formed the anionic spirocyclic Meisenheimer complex MC4 that shows clear changes in the absorption spectrum as well as negligible fluorescence emission (Figure 1b).

### 2.2. Using ZW1 or ZW3 for the Detection of F^−^ or CN^−^

As shown in Scheme 2, the pH-sensitivity of ZW1 (and ZW3) relies on the reactivity of the iminium proton, since it reacts with a base (e.g., tetrabutylammonium hydroxide), whereas the deprotonated imine form can react with an acid (e.g., perchloric acid in acetonitrile). The deprotonated imine group has the capability to quench the emission of the electron-poor 1,3,5-trinitrocyclohexadiene anion via photoinduced electron transfer. In light of these findings, we thought of using these compounds as chemosensors to detect other anions that are able to selectively deprotonate and/or interact with their iminium proton so as to activate the quenching mechanism of the fluorescence of their zwitterionic state. With this goal, we focused our attention on two main types of analytes: fluoride and cyanide anions.

**Figure 2:**
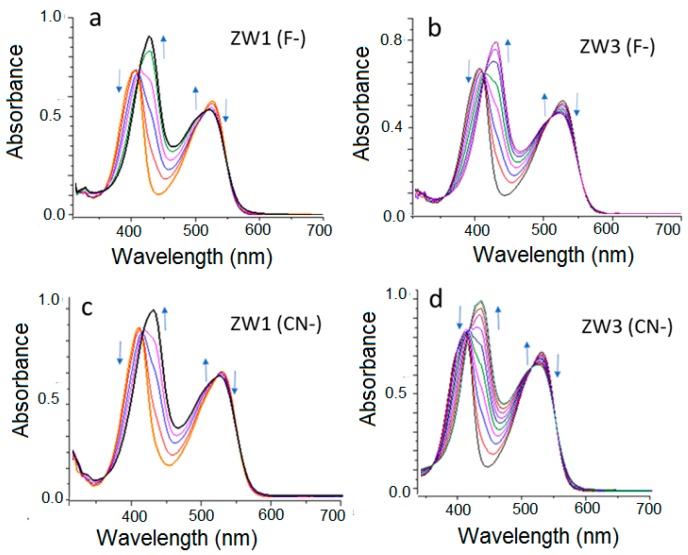
Spectrophotometric titrations (UV/vis absorption spectra) of ZW1 (left, [_ZW1_] ~ 5 × 10^−5^ M) and ZW3 (right, [_ZW3_] ~ 7 × 10^−5^ M) in acetonitrile with an acetonitrile solution (10 mM) of (**a**,**b**) tetraethylammonium fluoride and (**c**,**d**) tetraethylammonium cyanide. The arrows indicate the evolution of the absorption bands for anion/ZW molar ratios from 0 to 1.

When micromolar solutions of ZW1 and ZW3 in a polar aprotic solvent such as acetonitrile were titrated with micromolar solutions of either tetraethylammonium fluoride (TEAF) or tetraethylammonium cyanide (TEACN), a change in the absorption spectrum of the two spirocyclic compounds (Figure 2) was observed. Aside from a clear red-shift of the absorption band at 406 nm, the evolution of two isosbestic points at 415 and 510 nm was also observed for both compounds, which indicated the unimolecular conversion of ZW1 or ZW3 into a new compound. Noticeably, the absorption spectra of the final species obtained after titration of ZW1 or ZW3 with TEAF or TEACN resembled those of the deprotonated states MC2 and MC4 (Figure 1a,b). This may suggest that the detection of fluoride and cyanide anions proceeds through an acid-base reaction, where both F^−^ and CN^−^ act as bases under our experimental conditions.

**Figure 3:**
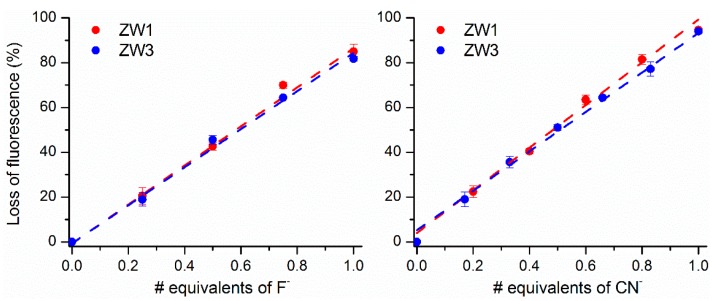
Standard curves for the fluorescence detection of fluoride and cyanide anions using acetonitrile solutions of ZW1 and ZW3 ([_ZW_] = 6 × 10^−6^ M), where the loss of emission intensity measured (λ_exc_ = 406 nm) upon controlled additions of up to 1 equivalent of tetraethylammonium fluoride (TEAF) and tetraethylammonium cyanide (TEACN) is plotted. Dots correspond to the average ΔF/F_0_ values determined after three independent experiments, while lines were obtained after linear fits of this data.

Moreover, colorimetric changes were accompanied by a decrease in fluorescence emission upon increasing the concentration of fluoride and cyanide anions. As shown in Figure 3, a linear response was observed in all the cases for the loss of fluorescence (ΔF/F_0_) of the initial ZW1 and ZW3 acetonitrile solutions upon the addition of TEAF or TEACN up to 1 mole equivalent, which resulted in a nearly complete emission “turn-off” of these compounds (~90%). Actually, the addition of a slight excess of cyanide and fluoride anions (~1.5 equivalents) totally switched off the fluorescence emission from ZW1 and ZW3 solutions.

**Figure 4:**
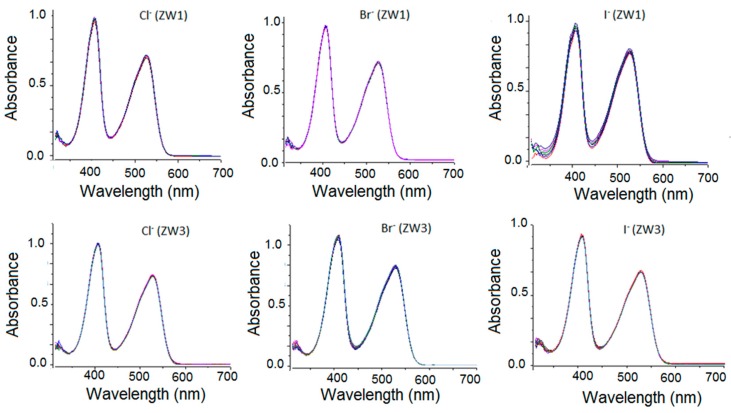
Spectrophotometric titrations (UV/vis absorption spectra) of ZW1 top three figures ([*_ZW_*_1_] ~ 6.0 × 10^−5^ M) and ZW3 bottom three figures ([*_ZW_*_3_] ~ 1.0 × 10^−4^ M) in acetonitrile with 10 mM acetonitrile solution*s* of tetrabut*y*lammonium chloride, tetrabut*y*lammonium bromide, and potassium iodide. The halide*/ZW* molar ratio for each measurement was 0, 0.25, 0.50, 0.75, 1, and 1.50. Note that no significant changes in the absorption spectrum were observed.

The selectivity of the colorimetric and fluorescence detection of fluoride and cyanide anions using ZW1 and ZW3 was investigated by exploring the effect of other anionic analytes of relevance: Cl^−^ (from tetrabutylammonium chloride), Br^−^ (from tetrabutylammonium bromide), I^−^ (from potassium iodide), H_2_PO_4_^−^ (from tetrabutylammonium dihydrogenphosphate), NO_3_^−^ (from potassium nitrate), and NO_2_^−^ (from potassium nitrite). The addition of up to 1.5 equivalents of these anions to solutions of ZW1 and ZW3 in acetonitrile did not result in measurable changes in the absorption spectrum of these zwitterionic compounds, as shown for some selected cases in Figure 4. Therefore, this demonstrates that ZW1 and ZW3 allow selective monitoring of F^−^ and CN^−^ based on colorimetric measurements.

**Figure 5:**
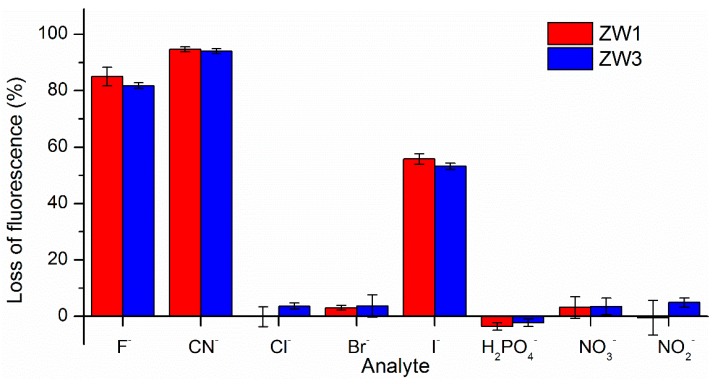
Histogram of the loss of fluorescence intensity registered for acetonitrile solutions of ZW1 and ZW3 ([_ZW_] = 6 × 10^−6^ M, λ_exc_ = 406 nm) upon the addition of 1 equivalent of different anions ([anion] = 6 × 10^−6^ M). The average of three independent experiments is plotted in each case.

In regard to fluorescence detection, Figure 5 shows the loss of emission intensity of ZW1 and ZW3 solutions in acetonitrile after the addition of 1 equivalent of all the anions of interest. Clearly, negligible effects in fluorescence emission were observed for chloride, bromide, dihydrogenphosphate, nitrate, and nitrite anions, in agreement with absorption spectra. This reveals that no interaction takes place between those analytes and ZW1 and ZW3, which can therefore selectively sense the presence of fluoride and cyanide anions. On the contrary, the addition of I^−^ did result in appreciable changes in fluorescence emission from the spirocyclic zwitterionic compounds, although no variation was observed in the absorption spectrum (see Figure 4). This clearly indicates that the interaction of iodide anions with ZW1 and ZW3 only occurs in the electronic excited state of these compounds, as expected due to the fluorescence quencher character reported for I^−^ when added over solutions of a large number of organic dyes [23]. Therefore, colorimetric characterization would be required to discriminate between cyanide or fluoride anions and iodide ions when using ZW1 and ZW3 as optical chemosensors.

**Figure 6:**
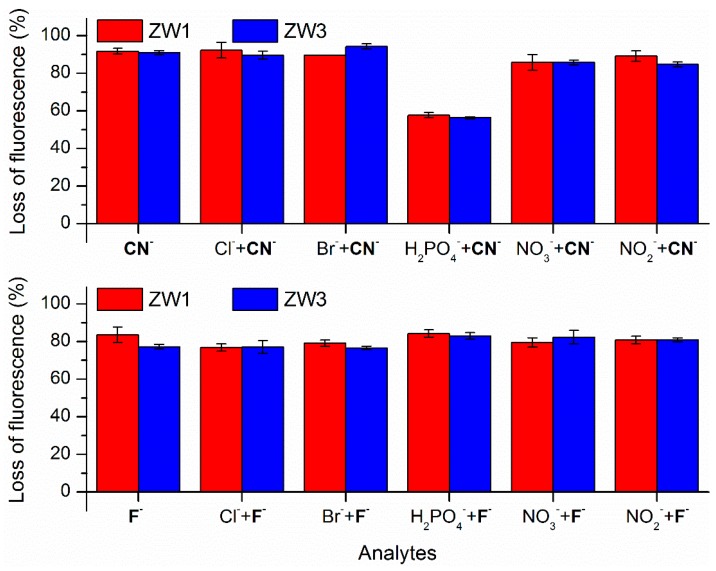
Histograms of the loss of fluorescence intensity registered for acetonitrile solutions of ZW1 and ZW3 ([_ZW_] = 6 × 10^−6^ M, λ_exc_ = 406 nm) upon the addition of 1 equivalent of F^−^ or CN^−^ ([F^−^] or [CN^−^] = 6 × 10^−6^ M ) and 1 equivalent of another anion ([_anion_] = 6 × 10^−6^ M). The average of three independent experiments is plotted in each case.

Finally, we wanted to explore the interference effects caused by the presence of additional ions when conducting the fluorescence detection of F^−^ and CN^−^ with ZW1 and ZW3. As shown in Figure 6, the presence of equivalent amounts of Cl^−^, Br^−^, NO_3_^−^, and NO_2_^−^ did not induce significant changes in the fluorescence response measured for fluoride and cyanide anions. Therefore, negligible interference effects were found in these cases for both ZW1 and ZW3. However, a different situation was encountered when fluorescence detection of F^−^ and CN^−^ was performed in acetonitrile media that also contained appreciable concentrations of H_2_PO_4_^−^. Under such circumstances, lower losses of emission intensity were observed for both ZW1 and ZW3 in the case of CN^−^ detection, whereas no changes in the fluorescence response were found for F^−^ sensing. This reveals selective interference effects induced by H_2_PO_4_^−^ anions when attempting the optical detection of cyanides. We ascribe this result to the presence of labile protons in H_2_PO_4_^−^, with which CN^−^ should interact in a similar manner as with ZW1 and ZW3. As such, cyanide anions will be involved in two competitive recognition processes and, consequently, their effect on the fluorescence signal of ZW1 and ZW3 will be lowered. On the other hand, the interaction of F^−^ with ZW1 and ZW3 must be much stronger than with H_2_PO_4_^−^, thereby accounting for the lack of interference effects observed in this case.

## 3. Discussion

In this work, we have explored the use of zwitterionic spirocyclic Meisenheimer complexes ZW1 and ZW3 as colorimetric and fluorescent chemosensors of fluoride and cyanide anions in solution. We have demonstrated that the absorption spectra of these compounds change upon the addition of F^−^ and CN^−^ ions, while their bright orange fluorescence switches off. Based on previous studies on the pH-dependence of the optical properties of ZW1 [11], the behavior observed herein should result from the interaction of the iminium proton of ZW1 and ZW3 with fluoride and cyanide ions, which must: (i) alter the absorption spectrum of the nearby 1,3,5-trinitrocyclohexadiene anion chromophore, as already observed upon deprotonation; and (ii) increase the electron density around the nitrogen atoms of the triazine ring so as to enable fluorescence quenching of the chromophore by photoinduced electron transfer. According to the concentration-dependent experiments shown in Figure 3, such an interaction between the iminium group of ZW1/ZW3 and F^−^/CN^−^ appears to follow a 1:1 stoichiometry and, in fact, complete inhibition of the emission from these fluorescent zwitterionic compounds was observed after the addition of a slight excess of fluoride and cyanide anions (~1.3 equivalents).

**Figure 7:**
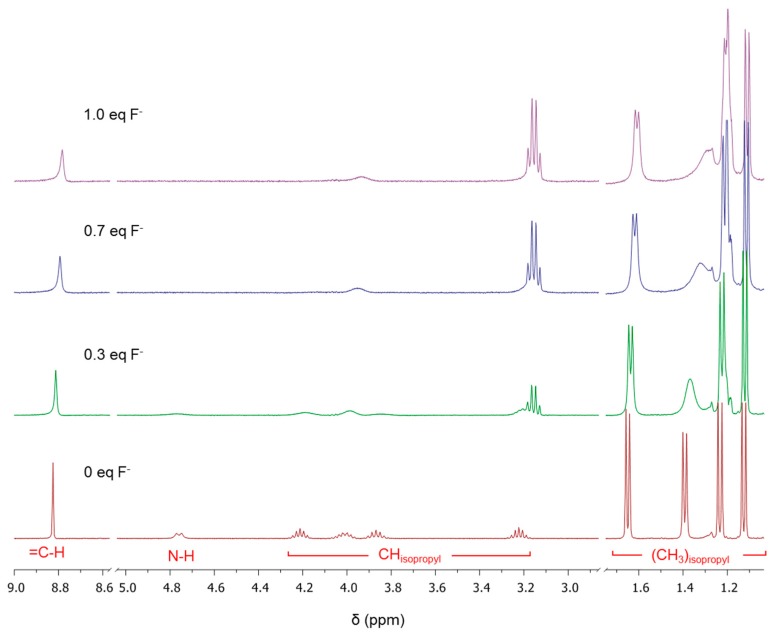
^1^H-NMR (360 MHz, CD_3_CN) spectra of ZW1 in solution upon the addition of 0, 0.3, 0.7, and 1.0 equivalents of fluoride anions. The increasing signals at δ ~ 3.2 and 1.2 ppm correspond to the ethyl and methyl protons of the tetraethylammonium counter-cations added. The same effects were observed for ZW3 solutions.

**Scheme 3:**
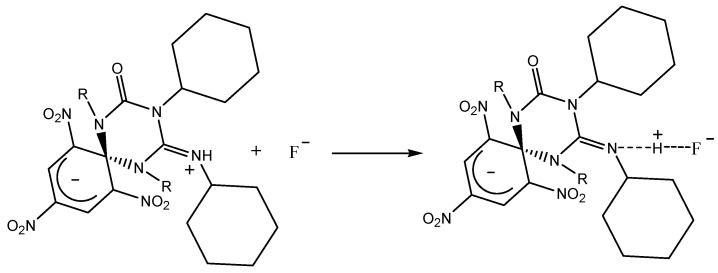
Plausible complexation between ZW3 and F^−^.

In the case of fluoride anions, molecular recognition by ZW1 and ZW3 could be ascribed to the selective complexation of a hard Lewis base such as F^−^ [24] with a hard Lewis acid (the iminium proton), thus providing an alternative way to induce the fluorescence “turn-off” process of zwitterionic spirocyclic Meisenheimer complexes without requiring OH^−^ addition (Scheme 3). To investigate this process, the titration of concentrated solutions of ZW1 and ZW3 in deuterated acetonitrile with fluoride anions was monitored by ^1^H-NMR (Figure 7). In these experiments, the addition of increasing amounts of F^−^ did not result in the appearance of a new set of signals corresponding to (ZW1---F^−^) and (ZW3---F^−^). Instead, the broadening and upfield shift of the initial ZW1 and ZW3 proton resonances was observed, from which we can only conclude that the interaction with fluoride anions is a highly dynamic process that cannot be resolved by ^1^H-NMR.

Similar results were obtained when investigating the interaction between ZW1 and ZW3 with CN^−^ by ^1^H-NMR, thus again revealing the fast dynamics of this process. In this case, however, a different type of interaction may take place owing to the soft Lewis base character of cyanide anions. A more plausible explanation would then be that CN^−^ acts as a rather strong Brönsted base in non-protic solvents such as acetonitrile, thereby deprotonating the iminium group of ZW1 and ZW3. Actually, cyanide (and also fluoride) anions are known to present K_b_ values in DMSO that are several orders of magnitude higher than those of other anions explored in this work (e.g., Cl^−^, Br^−^), as well as weak bases such as NH_3_ [25], which would justify their selective interaction with ZW1 and ZW3 via simple acid-base processes. According to this hypothesis, the distinct K_b_ values of CN^−^ and F^−^ in acetonitrile would account for the different interference effects measured for those analytes when an additional Brönsted acid such as H_2_PO_4_^−^ is present in the medium.

**Figure 8:**
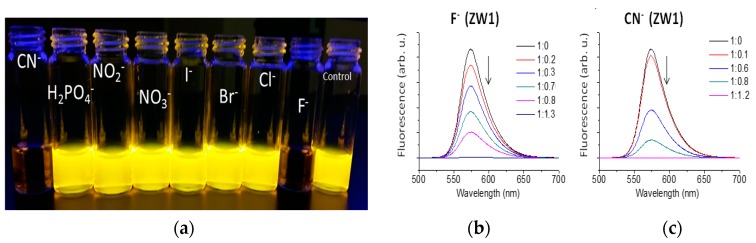
(**a**) Fluorescence response of ZW1 in acetonitrile upon the addition of different anions. Spectrophotometric fluorescent titrations of ZW1 in acetonitrile with a solution of tetraethylammonium (**b**) Fluoride (10 mM) and (**c**) Cyanide (10 mM) in acetonitrile. The arrows indicate the evolution of the emission band for anion/ZW1 molar ratios from 0 to 1 (fluorescence, [ZW1] = 3.0 × 10^−6^ M). Note that the equimolar addition of fluoride or cyanide results in the nearly complete suppression of the emission from the sample and, consequently, no fluorescence spectrum is visible in these cases in the pictures.

Regardless of the actual mechanism of interaction between ZW1/ZW3 and F^−^/CN^−^, our measurements have also demonstrated that zwitterionic spirocyclic Meisenheimer compounds can selectively recognize fluoride and cyanide anions with respect to other relevant analytes (Cl^−^, Br^−^, I^−^, H_2_PO_4_^−^, NO_3_^−^, NO_2_^−^) by means of colorimetric and fluorescence experiments, and with minimal interference effects in most cases (Figure 8). As a consequence, the development of ZW compounds provides a facile, fast, and cheap method for the optical detection and quantification of fluoride and cyanide anions.

## 4. Materials and Methods

All solvents used were distilled over CaH_2_ and stored over activated molecular sieves (3 Å). All chemicals used were of reagent grade with >98% purity and were used without further purification. Flash column chromatography was performed on silica gel 60 Å, particle size 35–70 μm. ^1^H-NMR spectra were recorded on Bruker DPX360 (360 MHz) (Billerica, MA, USA) and Bruker AV-III400 (400 MHz) spectrometers. ^13^C-NMR spectra were recorded on a Bruker AV-III400 (100 MHz) spectrometer with complete proton decoupling. Proton chemical shifts are reported in ppm (δ) (CDCl_3_, δ = 7.26 or CD_3_CN, δ = 1.94). Carbon chemical shifts are reported in ppm (δ) (CDCl_3_, δ = 77.2 or CD_3_CN, δ = 1.32) and the J values are reported in Hz. High resolution mass spectra (HRMS) were recorded on an ESI-QTOF Bruker Daltonics microTOF-Q spectrometer.

UV-Vis absorption spectra were recorded using a HP 8452A spectrophotometer (Agilent,) (Santa Clara, CA, USA) with Chemstation software. Emission spectra were recorded by means of a custom-made spectrofluorometer, where a cw diode laser (Z-laser, λ_exc_ = 532 nm) was used as an excitation source and the emitted photons were detected in an Andor ICCD camera coupled to a spectrograph. In all cases, spectroscopy quality solvents and 1-cm quartz cuvettes were used. Temperature was controlled using a refrigerated circulator bath (Huber MPC-K6) (Huntersville, NC, USA) connected to the sample holder. Fluorescence quantum yields were determined for highly diluted solutions of the compounds of interest to prevent self-absorption processes (absorption < 0.05 at the excitation wavelength), and they were measured relative to *N,N′*-bis(butyl)-1,6,7,12-tetra-(4-*tert*-butylphenoxy)perylene-3,4:9,10-tetracarboxylic diimide in CH_2_Cl_2_ (Φ_f_ = 1) [26]. Spectrophotometric and spectrofluorometric measurements of anion titration were conducted in the following way: a stock solution of ZW1 or ZW3 (1.00 mM) in acetonitrile (5 mL) was prepared. In a 1-cm path length quartz cuvette, the stock solution was combined with spectroscopic grade acetonitrile to give a maximum absorption below 1.0 at 406 nm and a total volume of 3.0 mL. A stock solution of the anion in acetonitrile (1.0 mM) was then injected at a 0.1 mole equivalent increment with a microsyringe.

### 4.1. Synthesis of ZW1

A solution of picric acid (3.0 g, 13.0 mmol) in acetonitrile (35.0 mL) was added slowly to a solution of *N,N′*-diisopropylcarbodiimide (8.0 g, 63.5 mmol, 4.8 equiv.) in acetonitrile (20 mL). The reaction mixture was stirred under a blanket of argon gas for 24 h at 60 °C. The organic solvent was removed under reduced pressure to obtain the crude product in the form of an orange powder. The crude product was dissolved in boiling methanol (120 mL), to which water (80 mL) was later added, and then the solution was cooled to 0 °C. Picric acid-free crude orange crystals (1.9 g) were recovered by filtration and consisted of a mixture of ZW1 and NA1. These products were separated by column chromatography (silica gel; ethyl acetate/hexane, 30:70). The first eluting component was NA1, which upon removal of the solvent gave yellow crystals (yield 0.8 g, 15.8%). The second eluting component was recovered after removing the eluting solvent under reduced pressure to give red crystals of ZW1 (yield 1.1 g, 15.5%). m.p. 192–195 °C with decomposition. Compound ZW1 was further purified by the addition of hexane to a saturated solution of ZW1 in ethyl acetate. Fine red crystals were recovered by suction filtration. 

Characterization of *ZW1*: ^1^H-NMR (360 MHz, CDCl_3_): δ = 1.11 (d, *J* = 6.9 Hz, 6H), 1.22 (d, *J* = 7.2 Hz, 6H), 1.38 (d, *J* = 6.3 Hz, 6H), 1.66 (d, *J* = 6.6 Hz, 6H), 3.24 (sept, *J* = 6.9 Hz, 1H), 3.87 (sept, *J* = 7.2, 1H), 4.21 (m, 1H), 4.74 (sept, *J* = 6.6 Hz, 1H), 4.30 (d, *J* = 6.9 Hz, 1H), 8.8 (s, 2H) ppm. ^13^C-NMR (75 MHz, CDCl3): δ = 19.90, 22.01, 23.11, 24.10, 51.05, 51.10, 53.12, 58.40, 82.28, 120.13, 125.54, 130.83, 145.01, 154.71 ppm. IR (KBr): ν˜ = 3449, 2978, 1709, 1586, 1523, 1488, 1316,1280, 1205, 1194, 1051 cm^−1^. HRMS (ESI-QTOF): *m*/*z* calculated for [C_32_H_47_N_7_O_7_ + H]: 482.2358; experimental: 482.2353. C_20_H_31_N_7_O_7_ (481.5): calcd. C 49.91, H 6.44, N 20.38; found, C 49.86, H 6.51, N 20.09. UV-Vis (ACN): λ^abs^_(max)_ = 406 nm (ε_abs_ = 16,600 M^−1^cm^−1^); fluorescence (ACN, λ_exc_ = 473 nm) λ_fl (max)_ = 563 nm, φ_fl_ = 0.5.

Characterization of *NA1*: M.p. 156–158 °C. ^1^H-NMR (360 MHz, CDCl_3_): δ (ppm) = 1.15 (d, *J* = 6.3 Hz, 6H), 1.17 (d, *J* = 6.6 Hz, 6H), 3.92–4.03 (m, 1H), 4.22 (sept, *J* = 6.9 Hz, 1H), 4.36 (d, *J* = 7.5 Hz, 1H), 8.87 (s, 2H) ppm. IR (KBr): ν˜ = 723, 912, 1360, 1555, 1611, 1655, 2864, 2940, 3102, 3320 cm^−1^. C_13_H_17_N_5_O_7_ (355.3): calcd. 43.95, H 4.82, N 19.71; found C 43.86, H 4.70, N 19.92. 

### 4.2. Synthesis of ZW3

This compound was synthesized following a similar procedure to the one used to synthesize ZW1, using *N,N′*-diciclohexylcarbodiimide (13.0 g, 63.5 mmol, 4.8 eq.) instead and catalytic amounts of *N,N*-dimethylaminopyridine. Compound ZW3 was obtained as red needles (2.33 g, yield 20%). M.p. 183–186 °C with decomposition.

Characterization of *ZW3*: ^1^H-NMR (360 MHz, CDCl_3_): δ (ppm) = 0.92–1.43 (m, 18H), 1.50–2.34 (m, 20H), 2.49 (q, *J* = 11.9 Hz, 2H), 2.64 (tt, *J* = 11.9, 3.6 Hz, 1H), 3.29 (m, 1H), 3.48 (m, 1H), 3.65 (tt, *J* = 11.7, 3.2 Hz, 1H), 4.53 (d, *J* = 9.0 Hz, 1H), 9.03 (s, 2H). IR (KBr): ν˜ = 3417, 2938, 2854, 1706, 1586, 1512, 1488, 1430, 1247 cm^−1^. HRMS (ESI-QTOF): *m*/*z* calculated for [C_32_H_47_N_7_O_7_ + H]: 642.3610; experimental: 642.3601. UV-Vis (ACN): λ^abs^_(max)_ = 406 nm (ε_abs_ = 10 810 M^−1^cm^−1^); fluorescence (ACN, λ_exc_ = 473 nm) λ_fl (max)_ = 563 nm, φ_fl_ = 0.6.

## 5. Conclusions

We synthesized two stable zwitterionic spirocyclic Meisenheimer compounds (ZW1 and ZW3) that show intense orange fluorescence in organic solvents such as acetonitrile. Their fluorescence could be “turned-off” upon the addition of c.a. 1 mole equivalent of F^−^ or CN^−^ anions, which was also accompanied by colorimetric changes. These effects were not observed for other anions such as Cl^−^, Br^−^, I^−^, NO_2_^−^, NO_3_^−^, or H_2_PO_4_^2−^, which makes ZW1 and ZW3 useful optical probes for the fast and direct detection and quantification of F^−^ and CN^−^.
